# Geospatial Assessment of Pesticide Concentration in Ambient Air and Colorectal Cancer Incidence in Arkansas, 2013–2017

**DOI:** 10.3390/ijerph19063258

**Published:** 2022-03-10

**Authors:** Lihchyun Joseph Su, Sean G. Young, Josephine Collins, Eryn Matich, Ping-Ching Hsu, Tung-Chin Chiang

**Affiliations:** 1 Department of Epidemiology, Fay W. Boozman College of Public Health, University of Arkansas for Medical Sciences, Little Rock, AR 72205, USA; ljsu@uams.edu; 2 Department of Environmental and Occupational Health, Fay W. Boozman College of Public Health, University of Arkansas for Medical Sciences, Little Rock, AR 72205, USA; ematich@uams.edu (E.M.); phsu@uams.edu (P.-C.H.); 3 Department of Psychology, Ouachita Baptist University, Arkadelphia, AR 71998, USA; col66266@obu.edu; 4 Department of Biochemistry & Molecular Biology, University of Arkansas for Medical Sciences, Little Rock, AR 72205, USA; tchiang@uams.edu

**Keywords:** pesticide, ambient, colorectal cancer, geospatial

## Abstract

Exposure to various agricultural pesticides has been linked to colorectal cancer (CRC), mostly among farmworkers and applicators. Given the potential pesticide drift in ambient air, residents near farmland may be exposed to carcinogenic pesticides even if they are not actively engaged in pesticide application. Pesticide air pollution at the county level was estimated using the 2014 National Air Toxics Assessment. CRC incidence data were acquired from the Arkansas Central Cancer Registry for 2013–2017. We ran ordinary least squares (OLS) regression models, finding significant spatial autocorrelation of residuals for most models. Using geographically weighted regression (GWR) we found age-adjusted CRC incidence rates vary in an increasing west-to-east gradient, with the highest rates in the Arkansas Delta region. A similar gradient was observed in the distribution of the population living below the poverty line and the population percentage of Black people. Significant associations between Trifluralin (crude model only), Carbon Tetrachloride, and Ethylene Dibromide with CRC incidence rates in OLS models only explained 5–7% of the variation and exhibited spatial autocorrelation of residuals. GWR models explained 24–32% (adjusted r^2^ 9–16%) of CRC incidence rate variation, suggesting additional factors may contribute to the association between pesticides and CRC.

## 1. Introduction

The overall mortality rate for invasive colorectal cancer (CRC) has steadily declined over the past decade. However, CRC remains the second most common cause of cancer-related deaths in the United States [1]. Similar to many other cancer types, there are some genetic mutations associated with CRC [2,3]. There are also many lifestyle [1] and environmental factors [4,5,6,7,8] that may enhance the likelihood of such cancers. It is still relatively unknown whether environmental exposures and/or lifestyle factors may exacerbate cancer outcomes among CRC patients.

The mortality rate of CRC remains the highest in the Mississippi (MS) River Delta, which includes the Arkansas (AR) Delta [9]. This cancer health disparity has been attributed to various factors, such as higher proportions of elderly residents, higher proportions of minorities, lower socioeconomic status, poor access to resources, and diet [1,9]. Agricultural pesticide exposure, including herbicides, insecticides, fungicides, and many others, is commonly thought to be linked to CRC risk [5,10,11]. Pesticide exposure can result from occupations relating to farming, pesticide application, and manufacturing. Additionally, spouses of farmers, those living in rural areas, those who eat non-organic foods, or those who apply or have pesticides applied to their yards can also be exposed [12,13,14].

Pesticides contribute to many types of pollution, including soil, water, and air pollution. The air can also be contaminated through pesticide drift. This phenomenon occurs because pesticides are emitted into the air when applied using a sprayer in a dry particle form [15]. Once pesticides enter the atmosphere, they can be moved through the air and across the land to areas where the pesticide was not applied [16]. This can include crops and fields that are near a pesticide application spot. When pesticides drift through the air and settle on food that was not a part of the original pesticide application, humans can be exposed to the pesticides from these contaminated crops [15,16].

The National Air Toxics Assessment (NATA) by the Environmental Protection Agency (EPA) provides a systematic evaluation of air toxics in the U.S. since 1996 [17]. Air toxics include hazardous air pollutants and diesel particulate matter. To date, there have been six versions of the NATA. The latest assessment occurred in 2014 and was released in 2018. The NATA provides a variety of information, including types of pollutants, sources of emissions, possible long-term health risks due to emission exposure, and cancer risk estimation based on breathing air toxics over many years. The NATA data are collected from the 48 contiguous states and include ambient air measurements for 131 chemicals aggregated to the county level. The 2014 NATA used in this study contains information for 180 air toxics and diesel particulate matter [17].

Exposure to air toxics is related to increased cancer incidence rates and other adverse health effects. The level to which individuals experience these health effects depends on the type and concentration of pollutants they are exposed to, the frequency and length of exposure, and their health characteristics. The NATA takes emissions data and estimates health risks based on these factors. Many pesticides included in this analysis are reasonably anticipated to be human carcinogens based on their relationship to increased cancer incidence and risk.

Exposure to various agricultural pesticides has been linked to CRC [18]. Given the potential pesticide drift in the ambient air, residents near farmland may be inadvertently exposed to carcinogenic pesticides even if they are not actively engaged in the pesticide application. By investigating the relationship between ambient air concentrations of pesticides and CRC incidence rates, the resulting information could help lead to policies concerning the methods of applying pesticides to reduce the risk of CRC. The investigated chemicals in this study focused on pesticides with known carcinogenic characteristics present in a recent review article that examined the relationship between pesticide exposure and CRC incidence rates [19]. We hypothesized that aggregated ambient air pesticide concentrations are associated with CRC incidence rates at the county level. We presented a novel geospatial assessment of the relationship between six specific pesticide chemicals found in ambient air in Arkansas and CRC incidence rates from 2013 to 2017.

## 2. Materials and Methods

We used the 2014 edition of the NATA released in 2018 to estimate air pollution from pesticides. The methodology used to gather and organize the NATA data is described in detail by the EPA [20]. Cancer incidence data from the AR Central Cancer Registry for the years 2013–2017 was used [21]. This dataset includes the number of cases, the crude cancer rate, and the age-adjusted cancer rate for all invasive cancers reported in AR, from which we extracted age-adjusted CRC incidence rates per 100,000. We acquired population characteristics at the county level from the U.S. Census Bureau and the American Community Survey [22], which included the population percentage over 65, percentage living under the poverty line, percentage of Black people, and percentage of males.

We mapped each of the 131 chemicals from the NATA at the county level for the state of AR using ArcGIS Pro mapping software (Esri, Redlands, CA, USA). Chemical concentrations were classified into five categories using Jenks natural breaks classification to visualize the distribution and gradients across the state. Following initial exploratory analyses and based on a recent review we conducted on the association between pesticides and CRC [19], we selected six chemicals for this study. The selected chemicals are commonly used as pesticides and in other agricultural activities. They are 2,4-dichlorophenoxyacetic acid (2,4-D), carbon tetrachloride, carbon disulfide, ethylene dibromide, methyl bromide, and trifluralin.

We performed ordinary least squares (OLS) regression for the selected chemicals and demographic variables with CRC incidence rate as the dependent variable. Spatial data commonly exhibit relationships that vary over space, such as positive associations in one area and negative associations in another. This is called nonstationarity. To address this, we further performed geographically weighted regression (GWR) in ArcGIS Pro. GWR creates a separate regression equation for each county, allowing the relationships between the pesticide, demographic, and cancer incidence to vary across the state, avoiding the problem of nonstationarity. For each model, we conducted Global Moran’s I tests of spatial autocorrelation on the model residuals. A statistically significant Global Moran’s I test result indicates spatial clustering of the errors, a potential indicator of model misspecification.

## 3. Results

**Figure 1:**
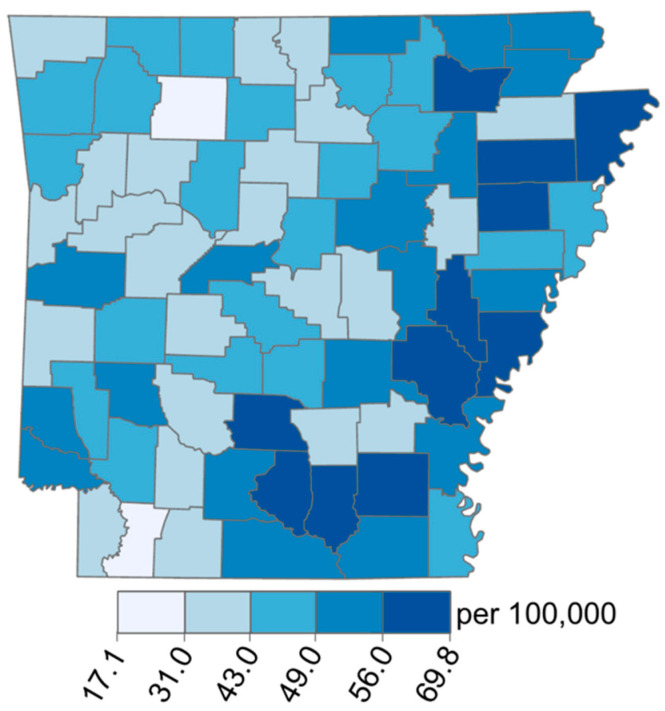
Map of age-adjusted colorectal cancer incidence by county in AR.

**Figure 2:**
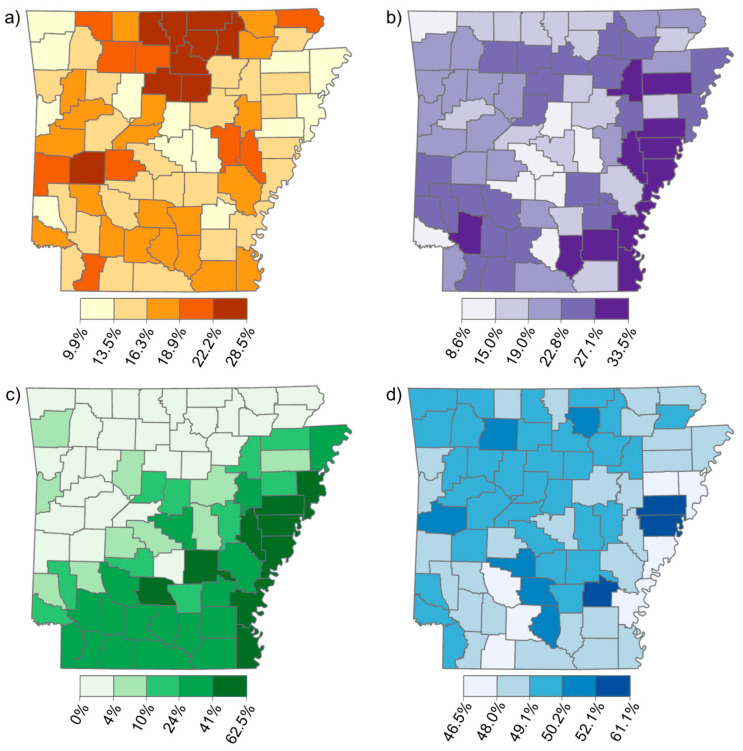
Maps of demographic variables as percentages of the population by county in AR: (**a**) Over age 65; (**b**) Living below the poverty line; (**c**) Black race; and (**d**) Male gender.

**Figure 3:**
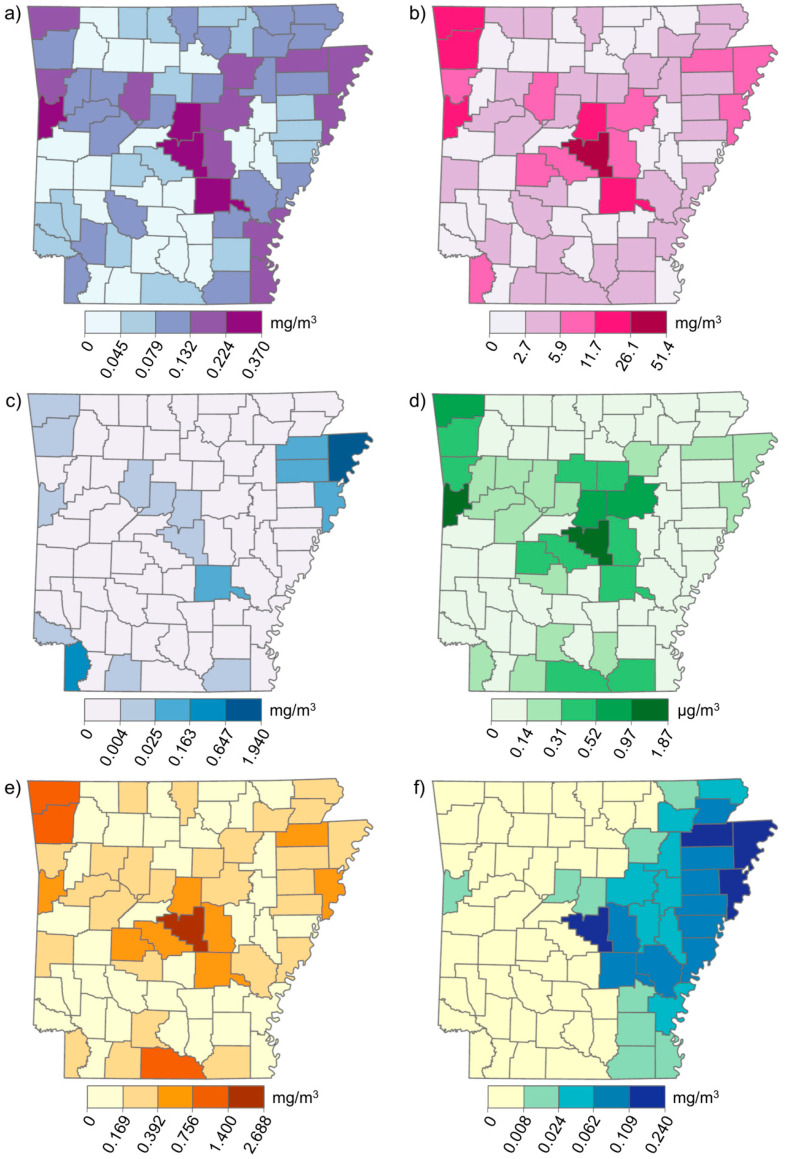
Distributions of selected chemicals’ ambient air concentrations by county in AR: (**a**) 2,4-D, salts and esters; (**b**) carbon tetrachloride; (**c**) carbon disulfide; (**d**) ethylene dibromide; (**e**) methyl bromide; and (**f**) trifluralin.

Age-adjusted CRC incidence rates vary across AR in an increasing west-to-east gradient, with the highest rates in the AR Delta region (Figure 1). A similar gradient is observed in the distribution of the population living below the poverty line (Figure 2b) and the population percentage of Black people (Figure 2c). The other two demographic factors, the population percentage over age 65, and the percentage of males (Figure 2a,d), did not appear to exhibit the same spatial pattern at the county level. Among the selected chemicals, only the distribution of trifluralin appeared to exhibit an obvious west-to-east increasing gradient when mapped using Jenks natural breaks (Figure 3f).

**Figure 4:**
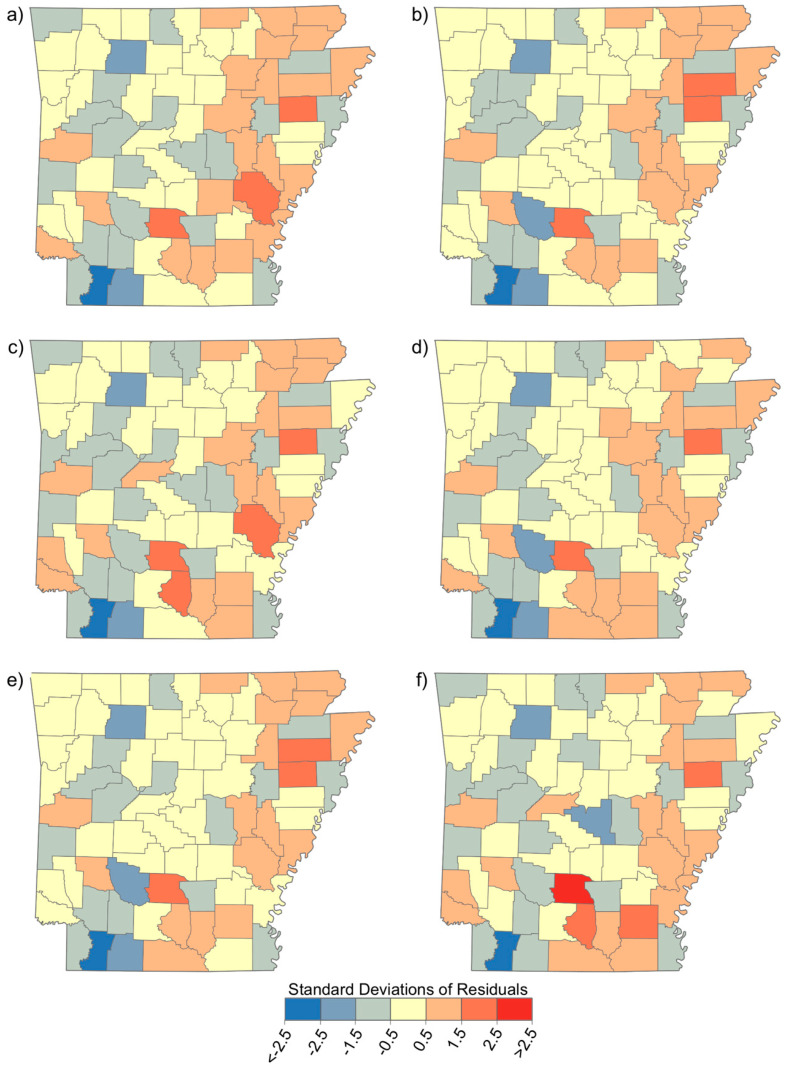
Distribution of residuals from Ordinary Least Squares regressions of selected chemicals, demographic variables, and colorectal cancer incidence: (**a**) 2,4-D, salts, and esters; (**b**) carbon tetrachloride; (**c**) carbon disulfide; (**d**) ethylene dibromide; (**e**) methyl bromide; and (**f**) trifluralin.

The OLS results are shown in Table 1, along with Global Moran’s I results. Trifluralin was significant (*p* = 0.03) in the crude model, yet only explained approximately 5% of the variation in CRC incidence rates at the county level; it was not significant in the adjusted model. Both Carbon Tetrachloride and Ethylene Dibromide were significant (*p* = 0.03 and *p* = 0.029 respectively) in the adjusted model, but similarly explained only about 7% of the variation in CRC incidence rates. The residuals from the OLS models are shown in Figure 4, where red represents underestimates and blue represents overestimates of CRC incidence rates. For all six chemicals, a gradient was apparent with overestimates more common in the western part of the state and underestimates more common in the eastern part of the state (i.e., in the AR Delta). This apparent spatial pattern of OLS residuals was confirmed with the Global Moran’s I test of spatial autocorrelation, which found significant (*p* < 0.05) spatial clustering of residuals in each chemical’s crude OLS model, and in four of the six adjusted models. Spatial clustering of OLS residuals indicates a spatial structure of the data not captured by the OLS model.

**Figure 5:**
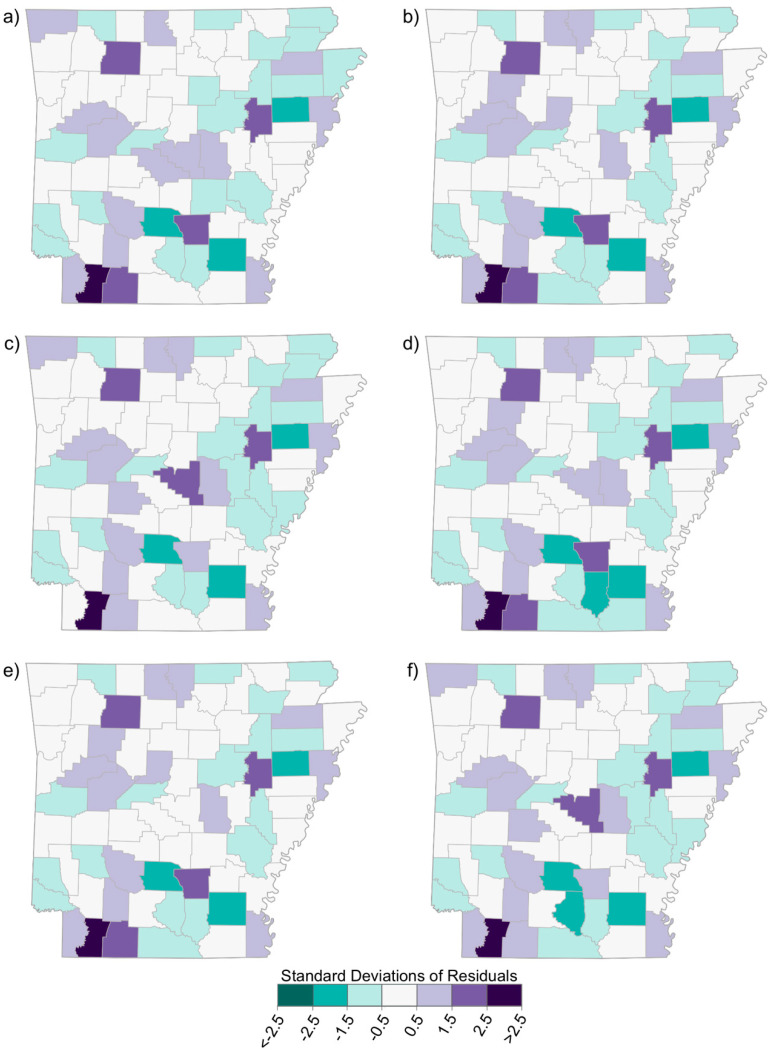
Distribution of residuals from geographically weighted regressions of selected chemicals, demographic variables, and colorectal cancer incidence: (**a**) 2,4-D, salts and esters; (**b**) carbon tetrachloride; (**c**) carbon disulfide; (**d**) ethylene dibromide; (**e**) methyl bromide; and (**f**) trifluralin.

GWR models were created for each chemical corresponding to the adjusted OLS models, resulting in R-squared values ranging from 0.244 to 0.318, and adjusted R-squared values ranging from 0.083 to 0.164 (Table 2). The GWR residuals are more randomly distributed when compared with the OLS residuals (Figure 5), confirmed by Global Moran’s I results.

## 4. Discussion

Each of the six selected pesticide chemicals exhibited a nonrandom spatial distribution in AR, as evidenced in the spatial patterns of the OLS residuals (Figure 4). These distributions violate the assumption of independence of observations, which partially explains the poor explanatory power of the OLS models, peaking at approximately 5% for trifluralin (see Table 1, Model 1). By allowing the regression equations to vary spatially with GWR, the problems of spatial autocorrelation of model residuals and nonstationarity were remedied and explanatory power grew substantially (ranging from 24.4 to 31.8%, see Table 2). This geospatial study examining the relationship between the concentration of several pesticides in the ambient air and CRC incidence suggests nonstationarity or relationships that vary over space. This means the relationship between a given pesticide, demographic factors, and CRC incidence partially depends on resident location rather than reflecting a constant and/or global linear relationship.

Heightened cancer mortality disparity in the AR Delta, particularly for CRC, has been reported since 2007 and has drawn the attention of both the scientific and media communities [1,9,23,24,25,26,27]. It has primarily been attributed to poverty and lack of CRC screening [9,24,25]. Significant efforts have been devoted to increasing CRC screening among Black populations in the AR Delta. We have observed a steady increase in CRC screening among Black populations based on the results from the Behavior Risk Factor Surveillance System telephone surveys [28]. However, the CRC mortality rate remained high in the region.

There has been considerable interest and concern about possible adverse health effects from persistent environmental pesticides as early as the 1960s [29]. A study compared the serum pesticide levels of adolescent CRC patients and their family members, from MS, AR, and Tennessee (TN) treated at St. Jude Children’s Hospital in Memphis, TN between 1974 and 1976 [30] to that of the general population in MS. They found blood pesticide levels on average among 10 out of 13 participants were not significantly higher than those of the MS general population. Two patients had exceptionally high levels of total dichloro-diphenyl-trichloroethane (DDT) (more than 200 parts per billion). One patient’s parents had significantly elevated levels of DDT. Other pesticides, such as dieldrin, β-hexachlorocyclohexane, and heptachlor, were examined but were not systematically higher than the comparison group. A few exceptional concentrations of these chemicals were reported among these adolescents or their family members. Serum pesticide residue levels are indexes for both intake and mobilization from adipose tissue stores. However, they may or may not reflect the levels of pesticides in the environment, the colon, or rectum, nor information on the route of exposure. Additionally, it is particularly worth mentioning that these pesticides were detectable among all patients and even among the comparison group. It is a concern how widespread the exposure to these pesticides was among the general population in the MS River Delta; thus, the comparison group used in this cross-sectional study may not have been appropriate.

Our group has recently reviewed the effect of the six pesticides examined for CRC [19]. Trifluralin is an herbicide that interrupts mitosis to prevent root development and is toxic to aquatic life because degradation results in different products. The International Agency of Research on Cancer (IARC) concluded in 1991 that, although trifluralin was found to be positively associated with non-Hodgkin’s lymphoma, there was insufficient evidence that trifluralin is a human carcinogen [31]. More recent reviews of the relationship between pesticide exposure and CRC concluded that a medium to large positive association exists between exposure to trifluralin and colon cancer [10,32,33]. A chlorophenoxy herbicide, 2,4-dichlorophenoxyacetic acid (2,4-D), is a growth regulator and is degraded in soil. Fertility problems in males have been related to 2,4-D, and the IARC has classified 2,4-D as a possible human carcinogen [34]. Two reviews that examined the association between specific pesticide exposure and CRC risk concluded that 2,4-D has a significant, medium to large positive association with colon cancer [10,33] and has also found to be significantly associated with rectal cancer [35]. This is of high concern because not only have both trifluralin and 2,4-D been shown to have a significantly positive association with colon and rectal cancers, but neither have been banned for agricultural use in the U.S. [36,37].

Carbon tetrachloride, ethylene dibromide, methyl bromide, and carbon disulfide are used as a fumigant in agriculture [38,39,40]. The major source of carbon tetrachloride in the air is industrial emissions [38]. It has been detected in surface water, groundwater, and drinking water as the result of agricultural activities. According to the evaluation by the IARC, there is insufficient evidence that tetrachloride is a human carcinogen, even though there is sufficient evidence in experimental animals.

Ethylene dibromide has been used as a scavenger for lead in gasoline, as a general solvent in waterproofing preparation, in organic synthesis, and as a fumigant for grain and tree crops [39]. An important but localized source of ethylene dibromide emissions is from grain and citrus fumigation centers and soil fumigation operations. There are many studies, both human and animal, evaluating the relationship between ethylene dibromide and cancer. Similar to carbon tetrachloride, the IARC concluded that there is insufficient evidence that ethylene dibromide is a human carcinogen, despite sufficient evidence in experimental animals. Methyl bromide is used as a soil and space (i.e., enclosed chamber) fumigant; as a pesticide on potatoes, tomatoes, and other crops; and as an extraction solvent for vegetable oil [40]. According to the IARC, there is insufficient evidence that methyl bromide is a human carcinogen, and there is limited evidence in experimental animals. Carbon disulfide was one of the first fumigants employed on a large scale. As far back as the 1800s in France, carbon disulfide was injected into the soil to control infesting insects at the roots of grapevines [41]. It was widely used as a soil or space fumigant and is used in areas with high temperatures, which favor the volatilization of the chemical. Carbon disulfide is toxic to humans, but the carcinogenic effect is inconclusive [19].

This geospatial study took advantage of the existing county-level cancer incidence data from the AR Central Cancer Registry, and the national ambient air toxic chemical data from EPA to generate a hypothesis regarding pesticide exposure and CRC risk. However, there are several limitations to this study’s approach. First of all, the ecologic study design used in this geospatial study examines the exposures and cancer outcomes at the aggregated population level. There is no information about exposure to pesticides studied by the individual, such that patients with cancer may or may not have been exposed to those specific pesticides. The concentration they were exposed to may depend on the type of occupation they held, their time spent outdoors, and household pesticides containing the same chemicals. Pesticide applicators and farmers are likely to have a higher level of exposure that cannot be distinguished in this study.

Additionally, the cancer incidence and ambient air toxic chemical data covered roughly the same time as the cancer incidence data, including a few extra years after the ambient air toxic chemical data sample collection. Cancer generally has a latent period from a few years to a few decades after the initial exposure. The timing of ambient air collection may be far removed from actual exposure unless exposure has been continuous. The lack of detailed, documented exposure history of the individual could further exacerbate the inadequacy of this geospatial examination. The cross-sectional nature of this study assumed exposure was consistent in the same geographic area. It cannot address the temporal relationship between pesticide exposure and CRC development. Finally, pesticide exposure may enter the human body through various routes, such as ingestion and dermal contact, in addition to inhalation from the ambient air. Although ambient air pesticide concentration may be a general indication for the use of pesticides in the region, it does not address pesticide exposure from other sources.

## 5. Conclusions

This study clearly identified a west-to-east increased pattern of CRC incidence in AR. We also observed similar patterns of increasing from west-to-east for the population percentage living under the poverty line, percentage of Black populations, and ambient air concentrations of trifluralin and 2,4-D. The results from the OLS and GWR models of pesticide chemicals and CRC incidence further the west-to-east geospatial pattern on all six chemicals examined. Both spatial autocorrelation and nonstationarity were detected, suggesting the need for GWR or similar spatial regression methods. However, even with GWR, only a relatively small percentage of the variance can be explained in the regression models (peaking at under 32% for Carbon Tetrachloride, adjusted r^2^ of 0.164, see Table 2). This moderate explanatory power suggests other individual-level factors may be more critical for the relationship between a given pesticide and CRC than a global linear relationship, or even county-specific relationships. Nevertheless, this geospatial examination offers preliminary support for collecting individual-level information on exposures, creating structured questionnaires, and exploring potential exposure biomarkers in epidemiologic studies in the future.

## Figures and Tables

**Table 1:** Ordinary least square regression for the relationship between selected agricultural chemicals and CRC at the county level. Global Moran’s I results of spatial autocorrelation on OLS residuals.

Chemical Name	Model 1 *				Model 2 *			
	Adj. R^2^	*p*-Value	Moran’s I	*p*-Value	Adj. R^2^	*p*-Value	Moran’s I	*p*-Value
2,4-D	−0.009	0.556	0.274	<0.001	0.031	0.164	0.148	0.021
Carbon Tetrachloride	0.015	0.152	0.267	<0.001	0.069	0.030	0.140	0.028
Carbon Disulfide	−0.003	0.375	0.294	<0.001	0.007	0.625	0.130	0.040
Ethylene Dibromide	0.023	0.102	0.251	0.001	0.070	0.029	0.123	0.050
Methyl Bromide	0.005	0.243	0.273	<0.001	0.055	0.056	0.138	0.030
Trifluralin	0.050	0.030	0.199	0.010	0.027	0.200	0.075	0.208

* Model 1: Crude model; Model 2: Adjusted for county population characteristics presented in Figure 2.

**Table 2:** Geographically weighted regression results for covariate-adjusted models, with Global Moran’s I results of model residuals.

Chemical Name	R^2^	Adj. R^2^	Moran’s I	*p*-Value
2,4-D	0.285	0.124	0.024	0.592
Carbon Tetrachloride	0.318	0.164	0.018	0.649
Carbon Disulfide	0.253	0.086	−0.005	0.903
Ethylene Dibromide	0.302	0.137	0.013	0.698
Methyl Bromide	0.309	0.148	0.022	0.611
Trifluralin	0.244	0.083	0.018	0.647

## Data Availability

NATA and ACS data are freely available from the EPA (https://www.epa.gov/national-air-toxics-assessment, accessed on 9 January 2022) and US Census Bureau (https://data.census.gov/, accessed on 9 January 2022) respectively. Cancer data from the AR Central Cancer Registry can be requested from the Arkansas Department of Health (https://www.healthy.arkansas.gov/programs-services/topics/arkansas-cancer-registry, accessed on 9 January 2022).
